# Nationwide Study of Neuropsychiatric Comorbidity and Medicines Use in Children With Autism Spectrum Disorder in Norway

**DOI:** 10.3389/fpsyt.2020.596032

**Published:** 2020-12-08

**Authors:** Yury Kiselev, Marte Handal, Vidar Hjellvik, Ted Reichborn-Kjennerud, Camilla Stoltenberg, Pål Suren, Alexandra Havdahl, Svetlana Skurtveit

**Affiliations:** ^1^Department of Life Sciences and Health, OsloMet – Oslo Metropolitan University, Oslo, Norway; ^2^Norwegian Institute of Public Health, Oslo, Norway; ^3^Institute of Clinical Medicine, University of Oslo, Oslo, Norway; ^4^Nic Waals Institute, Lovisenberg Diaconal Hospital, Oslo, Norway; ^5^Norwegian Centre for Addiction Research, University of Oslo, Oslo, Norway

**Keywords:** autism, children, psychotropic, CNS, medicines use

## Abstract

**Purpose:** Autism spectrum disorder (ASD) has a high rate of comorbidity. While many children with ASD are exposed to psychotropic medicines, their efficacy and safety in these patients are unclear. There is a need for more detailed knowledge on which medicines are most commonly used and for which disorders. We aimed to investigate (a) prevalence and incidence rate of ASD among Norwegian children, and further, among newly diagnosed ASD children in 2014, study the (b) co-occurrence of neuropsychiatric disorders, (c) use of psychotropic drugs, and (d) the relationship between co-occurring diagnoses and use of psychotropic drugs.

**Method:** Nationwide registry-based study of children 2–17 years old in Norway.

**Results:** The ASD prevalence was 0.76% and the incidence rate was 0.12% in 2014. Of the children who received an initial ASD diagnosis in 2014 (*n* = 1,234), 64.8% had one or more co-occurring neuropsychiatric diagnosis. Psychotropic medication use was moderate (~20% used stimulants or hypnotics) in general, and low in children without comorbidity (nearly only hypnotics). There was a good accordance between co-occurring diagnoses and indication for the prescribed medications.

**Conclusions:** Children with newly diagnosed ASD mainly received psychotropic drugs to treat co-occurring neuropsychiatric conditions.

## Introduction

Autism spectrum disorder (ASD) is a complex neurodevelopmental disorder, which is usually diagnosed at a young age. Deficits in social interaction and restrictive repetitive behaviors are considered to be the core diagnostic criteria (1). ASD is also associated with a plethora of other symptoms such as aggression, self-harm, irritability, hyperactivity, and many more. Reported prevalence of ASD varies significantly, and depends on region, size, age group, and study methodology (mostly parent questionnaires, insurance claims, and only few nation-wide registry based studies). Prevalence has been reported to be between ~0.3 and 2.8%, seemingly higher in the US than in the Nordic countries (2–10). Co-occurring psychiatric and neurological conditions are common among children with ASD. In a US study based on insurance claims data, anxiety disorders was present in as many as 17–30% of adolescents with ASD, epilepsy in 3–10%, ADHD in 47–51%, and even among 3–4 year olds up to 15% had two or more co-occurring disorders (11). A systematic review concluded that the median percentage of children (<12 years) and adolescents (12–17 years) with ASD using psychotropic medication is 41.9 and 42.5%, respectively (12). However, the percentage ranged from 3 to 80%. The most commonly used groups of medications include stimulants, antipsychotics, antidepressants and sleeping aids (13, 14). Unfortunately, most of the available studies of medication use have limitations like relying upon data from insurance claims or parent reports. No pharmacological treatments have demonstrated efficacy in reducing core symptoms of ASD, but the high prevalence of comorbidity might explain some of the psychotropic drug use reported in the literature.

In the current study we aim to use a nationwide Norwegian sample of children (2–17 years) diagnosed with ASD:

1. Estimate the prevalence (cumulative incidence) and incidence rate of ASD
2. Estimate the co-occurrence of psychiatric and neurological diagnoses;
3. Investigate the use of psychotropic drugs;
4. Explore the relationship between co-occurring diagnoses and psychotropic drug use.

## Materials and Methods

This study is based on data from the Norwegian Patient Registry (NPR), and the Norwegian Prescription Database (NorPD). Individual-level registry data from the NPR and the NorPD were linked using the unique (encrypted) personal identity number assigned to all individuals living in Norway.

### Data Sources

#### The Norwegian Patient Registry

The NPR is an administrative database of records reported by the secondary health care, i.e., all hospitals and outpatient clinics owned or reimbursed by the government, thus covering practically all children with psychiatric conditions. Thus, the NPR includes information on patients referred by a GP to secondary health care. The NPR has included unique personal identification numbers since 2008, and consequently the registry contains nationwide individual-level secondary health care data from 2008 and onwards. In this study, we used data from the period 2008–2015. Diagnoses in the NPR are coded according to the International Classification of Diseases, 10th revision (ICD-10). In the present study, the following diagnosis for ASD were identified: autistic disorder (F84.0), Asperger's syndrome (F84.5) and atypical or unspecified autism (AUA) (F84.1, F84.8, F84.9).

Co-occurring psychiatric diagnoses on mental, behavioral or neurodevelopmental disorders (all ICD F diagnosis) overall and specifically on hyperkinetic disorders (F90), behavioral and emotional disorders with onset usually occurring in childhood and adolescence (F91–98), mood [affective] disorders (F30–39), neurotic stress-related and somatoform disorders (F40–48), epilepsy (G40), and sleep disorders (G47) were obtained.

A validation study by Suren et al. indicated a high overall validity of ASD diagnoses assigned by secondary health care and confirmed that the technical aspects of the NPR data collection are functioning well (15).

#### The Norwegian Prescription Database (NorPD)

Information on dispensed psychotropic drugs to outpatients from all Norwegian pharmacies were drawn from the NorPD, which covers the entire Norwegian population (~5.4 million inhabitants) (16). In the present study, we included the patients' unique (encrypted) identity number, sex, age, the date of dispensing, and drug information [Anatomical Therapeutic Chemical (ATC) code]. Data on psychotropic drugs dispensed during a 365-day period after incident diagnoses for ASD in 2014 were included in the analyses. Incident diagnosis was used to ensure that the ASD diagnosis preceded our assessment of drug use. In the following, prescription drugs dispensed, as recorded in the NorPD, are referred to as drugs used, although we do not have information on actual compliance (17).

The following psychotropic drugs were investigated: stimulants (ATC code N06B); antiepileptics (N03A), antidepressants (N06A), antipsychotics (N05A), anxiolytics (N05B), hypnotics (N05C), alimemazine (R06AD01). Alimemazine is used in Norway as a hypnotic, particularly to children (18). Pain relievers as opioids (N02A), NSAIDS (M01A) and prescription paracetamol (N02BE01) were also investigated, but not presented in the tables because of low prevalence of use.

### Study Population

The study population consists of (a) all children aged 2–17 years in Norway in 2008–2014, (b) all children aged 2–17 years in Norway with incident diagnoses of ASD during in 2014. Information on birth year was acquired from NPR and used to calculate age in 2014.

### Analytical Approach

We estimated the *period prevalence (cumulative incidence)* of ASD diagnosis per 1,000 children aged 2–17 years during 2008–2014. Children or adolescents were included if they had been diagnosed with ASD at least once during the period 2008–2014. The denominator in the prevalence analyses was the total number of inhabitants in Norway in the different age groups per July 1st in 2014, as registered by Statistics Norway.

We identified individuals with an *incident diagnosis* of ASD in secondary health care in 2014. A diagnosis was defined as incident if an individual had not been registered in NPR with the diagnosis any of the six previous years back to January 1st 2008. The *incidence rate* was calculated as number of incident cases divided by the number of individuals in the population under risk.

We explored the proportion of ASD subtypes according to age group and gender for the individuals with incident diagnoses of ASD in the NPR in 2014. We calculated the proportion who got diagnoses for psychiatric and neurological comorbidities from 2008 to 2015. This long time interval was chosen in order to identify lasting comorbidities in children who are using secondary health care rarely. We also calculated the proportion who were dispensed psychotropic drugs during the 365-day period after the date of the first diagnosis for the same groups.

To explore the extent to which users of psychotropic drugs had diagnoses that might have been considered as indications for such use, we calculated the proportion of children with relevant diagnoses in the NPR for all users of stimulants, antiepileptics, antidepressants, and antipsychotics during 365-days after the first ASD diagnosis. The diagnoses were: for stimulants - hyperkinetic disorders (F90); for antiepileptics - epilepsy (G40); for antidepressants - mood [affective] disorders (F30–39) and neurotic, stress-related and somatoform disorders (F40–48), and for antipsychotics - psychotic and bipolar disorders (F20–29, F30, F31, F33.3).

### Ethical Considerations

The register-linkage was approved by The Regional Committee for Medical Research Ethics (2010/131) and by the Norwegian Data Protection Authority (10/00447-5).

## Results

### Periodic Prevalence (Cumulative Incidence) of ASD Diagnosis

The estimated prevalence of ASD in 2014 among 2–17-year-old children was 7.6 per 1,000 (95% confidence interval 7.4–7.8 per 1,000). Among 8-year old, the prevalence was 6.3 per 1,000 (5.7–6.9 per 1,000). The prevalence increased steadily with increasing age but leveled out at around 13 per 1,000 children at the age of 15–17 years. From the age of four, the prevalence was at least three times higher among boys than girls.

### Incidence of ASD Diagnosis in 2014

The incidence rate was 1.2 per 1,000 for 2–17-year-old children. In boys the incidence rate was 1.8 per 1,000 and in girls it was 0.6 per 1,000.

Table 1 shows the number of boys and girls aged 2–17, divided into three age groups, diagnosed with ASD in 2014 (*n* = 1,234), by ASD subtype. Asperger's syndrome was the most common diagnosis (*n* = 474), followed by autistic disorder (*n* = 415) and AUA (*n* = 345). For both Asperger's syndrome and AUA more children got the diagnosis with increasing age, while it was opposite for autistic disorder.

**Table 1:** Number of all 2–17 years girls and boys with incident diagnoses of autism spectrum disorders (ASD) (*N* = 1,234) in 2014 in Norway stratified on age.

**Autism spectrum disorder subtype (ICD-10 diagnosis)**	**Age group**	**Girls *N***	**Boys *N***	**Total *N***
**ASD total**	**2–17**	**308**	**926**	**1,234**
Autistic disorder (F84.0), *N* = 415	2–5	37	163	200
	6–11	21	115	136
	12–17	22	57	79
Asperger's syndrome (F84.5), *N* = 474	2–11^*^	31	147	178
	12–17	108	188	296
AUA (F84.1, F84.8, F84.9), *N* = 345	2–5	20	43	63
	6–11	23	109	132
	12–17	46	104	150

*Data from Norwegian Patient Registry*.

*AUA, atypical or unspecified autism*.

* *Combined age 2–11 are not shown due to privacy protection regulations*.

### Co-occurring Diagnoses in Children With Newly Diagnosed ASD

Nearly two thirds (64.8%) of the children with incident ASD diagnosis in 2014 also had another neuropsychiatric diagnosis, and the proportion of comorbidity was especially high (up to 71.9%) among children with Asperger's syndrome or AUA (Table 2).

**Table 2:** Number and percentage (%) of 2–17 old Norwegian girls and boys with an incident diagnosis of Autism spectrum disorders (ASD) in 2014 who had psychiatric and neurological comorbidities during period 2008–2015.

**Autism spectrum disorder subtype (ICD-10 diagnosis)**	**Psychotic disorders F20–29**	**Mood [affective] disorders (F30–39)**	**Neurotic, stress-related and somatoform disorders (F40–48)**	**Hyperkinetic disorders (F90)**	**Behavioral and emotional disorders (F91–98)**	**Epilepsy (G40)**	**Sleep disorders (G47)**	**Any ICD-10 F diagnosis except autism spectrum disorder**
**ASD total (*****N*** **=** **1,234)**	**15 (1.2)**	^*^(around 7)	^*^(around 12)	**311 (25.2)**	**272 (22.0)**	**92 (7.5)**	^*^(around 2)	**800 (64.8)**
**Autistic disorder (F84.0)**								
Girls (*N* = 80)	<4	<4	<4	8 (10.0)	10 (12.5)	10(12.5)	<4	42 (52.5)
Boys (*N* = 335)	<4	6 (1.8)	11 (3.3)	48 (14.3)	60 (17.9)	29 (8.7)	<4	185 (55.2)
**Asperger's syndrome (F84.5)**								
Girls (*N* = 139)	4 (2.9)	32 (23.0)	42 (30.2)	45 (32.4)	31 (22.3)	8 (5.8)	4(2.9)	100 (71.9)
Boys (*N* = 335)	4 (1.2)	30 (9.0)	45 (13.4)	108 (32.2)	89 (26.6)	11 (3.3)	10 (3.0)	227 (67.8)
**AUA (F84.1,F84.8, F84.9)**								
Girls (*N* = 89)	<4	8 (9.0)	14 (15.7)	23 (25.8)	24 (27.0)	10 (12.2)	4 (4.5)	64 (71.9)
Boys (*N* = 256)	4 (1.6)	11(4.3)	31 (12.1)	79 (30.9)	58 (22.7)	24 (9.4)	10 (3.9)	182 (71.1)

*Data from the Norwegian Patient Register (NPR)*.

*<4 denotes fewer than four individuals in the group. Exact numbers are not shown due to privacy protection regulations*.

* *Exact numbers are not shown due to privacy protection regulations*. *AUA, atypical or unspecified autism*.

Among girls who received an autistic disorder diagnosis, epilepsy and behavioral/emotional disorders were the two most common co-occurring diagnostic groups: each of the diagnostic groups had prevalence of 12.5%. In boys with an autistic disorder diagnosis, behavioral and emotional disorders and hyperkinetic disorder were most common: 17.9 and 14.3%, respectively.

Co-occurring diagnoses were common for both girls and boys with Asperger's syndrome. Among girls, the dominant diagnoses were hyperkinetic, neurotic/mood, and behavioral/emotional disorders (32.4–22.3%). The two most common conditions among boys were hyperkinetic (32.2%) and behavioral/emotional disorders (26.6%). It was among patients with Asperger's syndrome we saw the most pronounced differences between girls and boys: neurotic disorders were diagnosed in 30.2 vs. 13.4%, and mood disorders in 23.0 vs. 9.0%, respectively.

Neuropsychiatric disorders among children diagnosed with AUA were also common. The same conditions were most common among girls and boys: behavioral/emotional disorders (27.0 vs. 22.7%) and hyperkinetic disorders (25.8 vs. 30.9%).

### Psychotropic Drug Use in Children With Newly Diagnosed ASD

Children with autistic disorder were mainly prescribed hypnotics (including alimemazine): 16.3 of girls and 17.3% of boys (Table 3).

**Table 3:** Number and percentage (%) of 2–17 old Norwegian girls and boys with incident diagnoses of Autism spectrum disorders (ASD) in 2014 that were treated with CNS active drugs in the period of 365 days after the first diagnosis.

**Autism spectrum disorder subtype (ICD-10 diagnosis)**	**Stimulants *N* (%)**	**Antiepileptics *N* (%)**	**Antidepressants *N* (%)**	**Antipsychotics *N* (%)**	**Anxiolytics *N* (%)**	**Hypnotics/alimemazine^**a**^*N* (%)**	**Any of the drug groups**
**ASD total (*****N*** **=** **1,234)**	**215 (17.4)**	**58 (4.7)**	^*^(around 5)	^*^(around 5)	**36 (2.9)**	**236 (19.1)**	**442 (35.8)**
**Autistic disorder (F84.0)**							
Girls (*N* = 80)	6 (7.5)	7 (8.8)	<4	<4	4 (5.0)	13 (16.3)	18 (22.5)
Boys (*N* = 335)	30 (9.0)	17 (5.1)	<4	11 (3.3)	8 (2.4)	58 (17.3)	88 (26.3)
**Asperger's syndrome (F84.5)**							
Girls (*N* = 139)	35 (25.2)	7 (5.0)	23 (16.5)	14 (10.1)	6 (4.3)	39 (28.1)	78 (56.1)
Boys (*N* = 335)	78 (23.3)	5 (1.5)	24 (7.2)	15 (4.5)	4 (1.2)	64 (19.1)	133 (39.7)
**AUA (F84.1, F84.8, F84.9)**							
Girls (*N* = 89)	15 (16.9)	8 (9.0)	6 (6.7)	2 (2.2)	4 (4.5)	17 (19.1)	32 (36.0)
Boys (*N* = 256)	51 (19.9)	14 (5.5)	7 (2.7)	16 (6.3)	10 (3.9)	45 (17.6)	93 (36.3)

*Data from the Norwegian Patient Register (NPR) and the Norwegian prescription database (NorPD)*.

a *Alimemazine is used in Norway as a hypnotic*.

*<4 denotes fewer than four individuals in the group. Exact numbers are not shown due to privacy protection regulations*.

* *Exact numbers are not shown due to privacy protection regulations*.

*AUA, atypical or unspecified autism*.

Children with Asperger's syndrome were mainly prescribed stimulants (25.2 of girls and 23.3% of boys) and hypnotics (25.2 of girls and 18.2% of boys). There was a particular disparity in the use of antidepressants: these were prescribed to 16.5% of girls and only 7.2% of boys.

Children with AUA were mainly prescribed stimulants (16.9 of girls and 19.9% of boys) and hypnotics (including alimemazine) (19.1 of boys and 17.6% of girls).

Prescription drugs most commonly combined were stimulants and hypnotics, used by 83 (6.7%) of children with incident ASD.

The children who had ASD in the absence of any of the studied neuropsychiatric comorbidities received very little psychotropic drugs in general. Hypnotic drugs and alimemazine were the only drugs used to some extent by these children; around 13% were treated with hypnotic drugs (mostly melatonin), independent of ASD subtype and gender.

### Accordance Between Drug Use and Co-occurring Conditions in Children With Newly Diagnosed ASD

**Figure 1:**
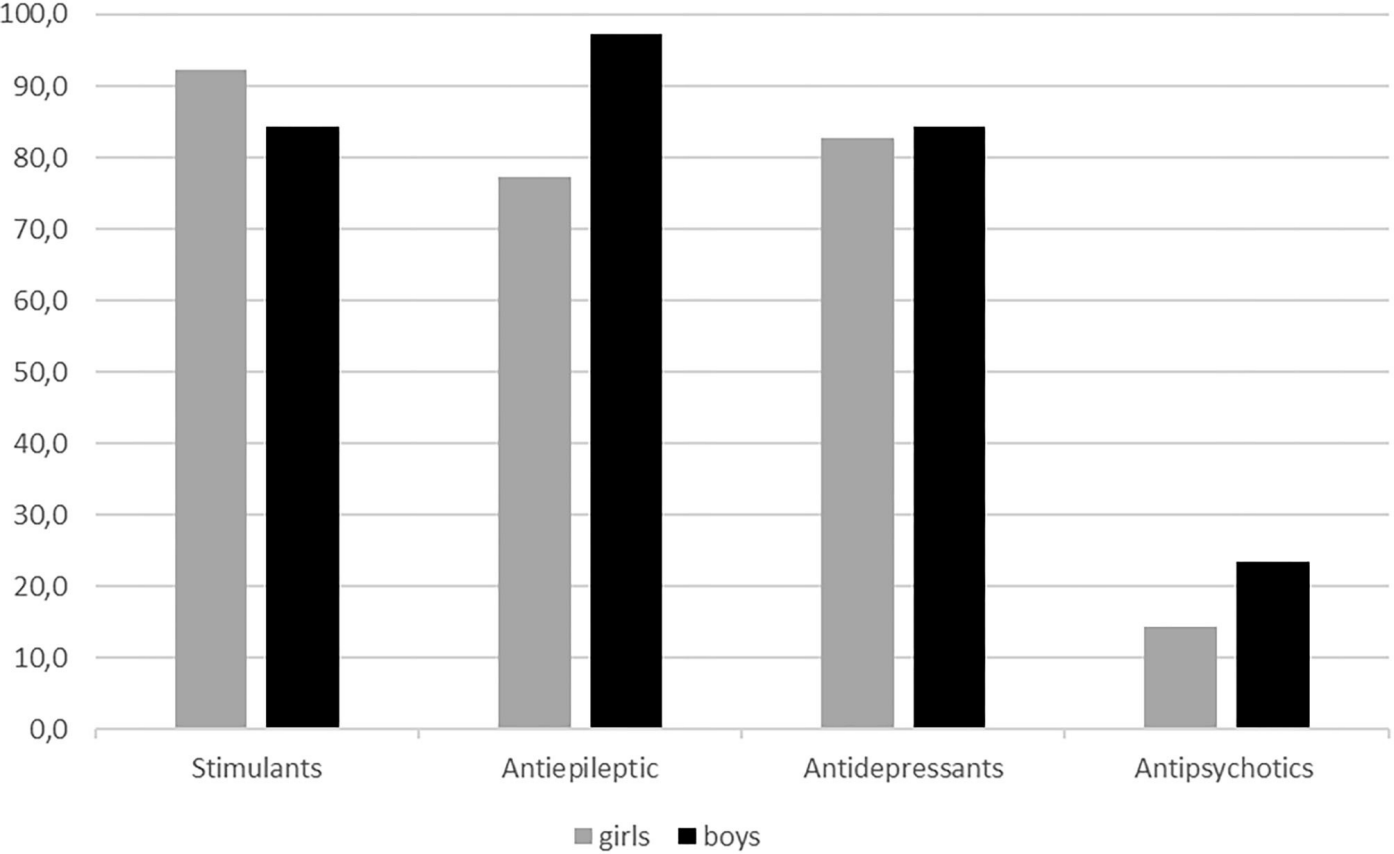
Accordance (%) between medication use and diagnosis stratified on gender.

There was a relatively good match between prescription of stimulants/ADHD drugs, antiepileptic drugs, and antidepressants, and co-occurring diagnoses for which these drugs are indicated (Figure 1). Of boys with an ASD diagnosis who were prescribed antiepileptic drugs, 94.6% had a diagnosis of epilepsy and 98.1% of boys who were prescribed stimulants had a diagnosis of ADHD. The corresponding proportions for girls were 77.3 and 94.4%. Most of the girls who received an antiepileptic drug because of a condition other than epilepsy had a mood or a pervasive and specific developmental disorder. In contrast, only a small proportion of children who received treatment with antipsychotic drugs had a diagnosis where such treatment is indicated (14.3% of the girls and 23.5% of the boys who were treated with antipsychotics had a psychotic condition).

## Discussion

### Main Findings

In this nation-wide study, we have investigated co-occurring diagnoses and use of psychotropic medication among children with ASD in Norway. Most children had one or more neuropsychiatric diagnosis in addition to ASD. Use of psychotropic drugs was common and similar to the UK, yet lower than in the US (19). There was high accordance between psychotropic drug prescriptions and co-occurring conditions where the drugs were indicated, suggesting that the high prevalence of psychotropic drug use mainly was associated with the high co-occurrence of neuropsychiatric conditions. Noteworthy, among ASD patients without a co-occurring condition, use of psychotropic drugs was low and consisted predominantly of hypnotics.

### Prevalence

We have estimated that the periodic prevalence (cumulative incidence) of ASD in 2–17-year-old children in 2008–2014 was 7.6 per 1,000. This is in line with previous studies from Norway (20). Periodic prevalence of ASD among 8-year old was 6.3 per 1,000, which is almost three times lower than in the recent Center for Disease Control (CDC) study from the US (6). The discrepancy between the US and Norwegian estimates might be due to differences in diagnostic approaches, systems – DSM and ICD and differences in methodological approaches, respectively. However, a study from four European countries showed a variation in prevalence among 8 years old children ranging from 4.8 in a South-East France to 31.3 per 1,000 in Iceland (21). Finland and South-West France had very similar prevalence estimates to our study, respectively, 7.7 and 7.3 per 1,000. Predominance of boys over girls in ASD prevalence is consistent with the globally observed pattern (6, 21–23).

### Co-occurring Diagnoses

It is important to be aware of potential neuropsychiatric comorbidity among children with ASD to ensure adequate treatment, and our estimate of 64.8% is consistent with recent findings (24–26). Previous studies have mostly investigated either small cohorts or parent-reported diagnoses, or did not provide numbers specifically for children, or measured symptoms of neuropsychiatric comorbidities rather than registered diagnoses. Although we have used a different approach, our results were overall in agreement with analyses published by others (26–29). Regarding psychotic diagnoses in children with ASD, one study reported these to be below 1% (29). Interestingly, ADHD prevalence seems to be lower in our ASD cohort than in the US-based studies—probably due to differences in diagnostic approach. The use of a national registry has allowed us to study the prevalence of neuropsychiatric comorbidity in the whole ASD population and look specifically into each of three main sub-diagnoses of ASD. Co-occurring conditions were particularly common among adolescents with Asperger's syndrome and AUA, and in the former group we observed a high share of girls with co-occurring neurotic and mood disorders. It remains to be seen whether this difference may be a consequence of underdiagnosing among boys.

### Use of Psychotropic Drugs

No pharmacological treatments have demonstrated efficacy in reducing core symptoms of ASD, and the previously reported high use of psychotropic medication in this group therefore requires further investigations. There is a lack of evidence supporting the benefit of most psychotropic medications in children with ASD, while adverse drug effects are common (10, 12, 30, 31). The safety of medication use in children with ASD is an important consideration, especially since the communication deficits inherent in ASD can impair their ability to verbalize adverse drug effects (32). Importantly, many countries (including Norway) lack national guidelines on treatment of children diagnosed with ASD, and choice of therapy is often based on expert opinions or local experiences. The high prevalence of comorbidity might explain some of the reported psychotropic drug use. Problematic behaviors and associated symptoms may also require use of medications. We observed very low use of psychotropic drugs in ASD-diagnosed children without co-occurring conditions. The only exception was hypnotics (mainly melatonin), which were used by 10.6% - this is not in contradiction to guidelines (31). Others have also reported that ASD patients without co-occurring conditions use less medicines than those with comorbidities (13).

For children with co-occurring disorders there is some evidence base for use of melatonin, risperidone, aripiprazole, methylphenidate, and atomoxetine (31). However, use of risperidone and aripiprazole is associated with significant adverse drug reactions (12). Our study identified hypnotics as the predominant medication group for ASD patients having co-occurring diagnoses, especially for patients with autistic disorder. Stimulants were most commonly used by patients with Asperger's syndrome and AUA, followed by hypnotics. Children with Asperger's syndrome also had prescriptions for antidepressants, especially girls. Use of hypnotics was probably driven by symptoms of anxiety, aggression and irritability in ASD patients, while stimulants and antidepressants might have been prescribed to control co-existing conditions—ADHD and depression. We observed a very low use of antipsychotics (4.8%), apart from girls with Asperger's syndrome (10.1%), in contrast to data on median prevalence of use 8.4–57.4% in a recent meta-analysis (12).

### Accordance Between Drug Use and Diagnoses

Unwarranted use of medications should be avoided particularly among children with ASD as no drugs prove to be effective against core symptoms of the disorder. Children with ASD often present a complicated mosaic of symptoms reflecting both core diagnostic criteria of ASD, associated symptoms and behaviors, and symptoms of the co-existing neuropsychiatric conditions. Use of psychotropic medications is therefore often difficult to attribute to one particular diagnosis, representing a challenge for pharmacoepidemiological studies.

For stimulants, antiepileptics, and antidepressants we observed a good agreement between prescriptions and diagnoses/indications. A moderate disagreement between use of antiepileptics and diagnosis of epilepsy among girls is possibly explained by presence of non-epilepsy indications, such as anxiety or depression. In the case of antidepressants, the observed disagreement is possibly due to use of these drugs to control repetitive behaviors, or non-core symptoms of ASD. The largest discrepancy between medication use and indication was registered for antipsychotics. We believe that the prescriptions of antipsychotics are driven by the desire to control aggression, self-harm and other non-core symptoms of ASD. Evidence of efficiency of antipsychotics in treating irritability in ASD exists only for specific drugs like risperidone and aripiprazole (31).

Although most of the children who were prescribed medications had co-occurring diagnoses for which these medications are indicated, this does not necessarily mean that the medication use was appropriate. There is a lack of consensus on best practice for diagnosing co-occurring disorders in ASD and findings suggest that many co-occurring diagnoses provided by clinicians are not supported by standardized diagnostic tools (33, 34). Furthermore, children with ASD may respond differently to medications than children without ASD. For example, whereas selective serotonin reuptake inhibitors (SSRIs) have demonstrated efficacy in children with obsessive-compulsive disorder, SSRIs do not appear to reduce obsessive-compulsive symptoms in children with ASD (35). Methylphenidate has shown efficacy in treating ADHD in children with ASD, but was less effective and had more side effects than in children with ADHD alone (36).

### Methodological Considerations

A major strength in this study is the use of national registries, which eliminates poor recall and minimizes selection bias. It also allows for linkage of data from the NPR and the NorPD on an individual level.

One limitation is that the NPR has individual level data only from 2008. Our estimation of periodic prevalence (cumulative incidence) may be an underestimation of the lifetime prevalence of ASD in the children who were 6 year and older in 2014 because we lack information about them the first years of their lives. However, as shown in Suren et al. (15), the recapture of ASD cases in consecutive years in NPR are high and therefore we believe that the predicted prevalence is quite similar to lifetime prevalence of ASD. Further the incident cases may not be truly incident cases. Given the restriction to incident ASD diagnoses across the age range of 2 to 17 years, our sample is likely to include a higher proportion of late diagnosed individuals. Another limitation is that we have no information about drugs administered to hospitalized children. However, in Norway, very few children stay in institutions for long periods, and certainly not for ASD.

A limitation in using NorPD data on medications is that the registry only include information on whether a drug has been dispensed, but not whether the medication is actually being used.

## Conclusion

Use of psychotropic drugs is common among children with ASD and co-occurring neuropsychiatric diagnoses in Norway, and most children using medications have been diagnosed with a co-occurring condition for which the drug is indicated. Medication use among children without co-occurring neuropsychiatric conditions is very low. There is a need for studies of efficacy and safety of CNS active medication use among children with ASD.

## Data Availability Statement

The datasets generated during and/or analyzed during the current study are not publicly available due to the restrictions inferred by Norwegian legislation for privacy protection, but anonymised data from this study can be made available upon reasonable request to the corresponding author. Requests to access these datasets should be directed to Yury Kiselev, dr.yurykiselev@gmail.com.

## Author Contributions

YK conceptualized the study, interpreted results, drafted the initial manuscript, and reviewed and revised the manuscript. VH obtained the data, interpreted results, and reviewed and revised the manuscript. SS conceptualized and designed the study, analyzed the data, interpreted results, drafted the initial manuscript, and reviewed and revised the manuscript. MH conceptualized and designed the study, drafted the initial manuscript, interpreted results, and reviewed and revised the manuscript. PS and AH conceptualized the study, drafted the initial manuscript, interpreted results, and reviewed and revised the manuscript. TR-K and CS conceptualized and designed the study, interpreted results, and reviewed and revised the manuscript. All authors contributed to the article and approved the submitted version.

## Conflict of Interest

The authors declare that the research was conducted in the absence of any commercial or financial relationships that could be construed as a potential conflict of interest.
